# Prediction of diabetes disease using an ensemble of machine learning multi-classifier models

**DOI:** 10.1186/s12859-023-05465-z

**Published:** 2023-09-12

**Authors:** Karlo Abnoosian, Rahman Farnoosh, Mohammad Hassan Behzadi

**Affiliations:** 1 Department of Statistics, Science and Research Branch, Islamic Azad University, Tehran, Iran; 2 School of Mathematics, Iran University of Science and Technology, Tehran, Iran

**Keywords:** Diabetes disease prediction, Machine learning classifiers, Ensemble machine learning models, Decision tree, Random forest, Feature selection

## Abstract

**Background and objective:**

Diabetes is a life-threatening chronic disease with a growing global prevalence, necessitating early diagnosis and treatment to prevent severe complications. Machine learning has emerged as a promising approach for diabetes diagnosis, but challenges such as limited labeled data, frequent missing values, and dataset imbalance hinder the development of accurate prediction models. Therefore, a novel framework is required to address these challenges and improve performance.

**Methods:**

In this study, we propose an innovative pipeline-based multi-classification framework to predict diabetes in three classes: diabetic, non-diabetic, and prediabetes, using the imbalanced Iraqi Patient Dataset of Diabetes. Our framework incorporates various pre-processing techniques, including duplicate sample removal, attribute conversion, missing value imputation, data normalization and standardization, feature selection, and k-fold cross-validation. Furthermore, we implement multiple machine learning models, such as k-NN, SVM, DT, RF, AdaBoost, and GNB, and introduce a weighted ensemble approach based on the Area Under the Receiver Operating Characteristic Curve (AUC) to address dataset imbalance. Performance optimization is achieved through grid search and Bayesian optimization for hyper-parameter tuning.

**Results:**

Our proposed model outperforms other machine learning models, including k-NN, SVM, DT, RF, AdaBoost, and GNB, in predicting diabetes. The model achieves high average accuracy, precision, recall, F1-score, and AUC values of 0.9887, 0.9861, 0.9792, 0.9851, and 0.999, respectively.

**Conclusion:**

Our pipeline-based multi-classification framework demonstrates promising results in accurately predicting diabetes using an imbalanced dataset of Iraqi diabetic patients. The proposed framework addresses the challenges associated with limited labeled data, missing values, and dataset imbalance, leading to improved prediction performance. This study highlights the potential of machine learning techniques in diabetes diagnosis and management, and the proposed framework can serve as a valuable tool for accurate prediction and improved patient care. Further research can build upon our work to refine and optimize the framework and explore its applicability in diverse datasets and populations.

**Supplementary Information:**

The online version contains supplementary material available at 10.1186/s12859-023-05465-z.

## Introduction

Chronic illness is a disease or condition that is ongoing or the effects of which are permanent [1, 2]. However, the consequences of this type of disease can have various negative effects on the quality of life, and a large part of the national budget is spent on chronic diseases [3, 4]. Diabetes is one of those chronic diseases that pose a major health risk and the number of medical causes of death is increasing every year, making it one of the biggest problems in emerging and developed countries [5, 6] High blood sugar levels have been linked to diabetes. The hormone insulin, which causes glucose from the food that enters the body to enter the bloodstream, is produced by one of the types of beta cells in the pancreas. Diabetes is caused by a lack of this hormone [7]. This disease can increase thirst, hunger, heart disease, kidney disease, etc., and even lead to the death of the patient [8, 9]. Diabetes can be divided into two types: type 1 and type 2. Patients with type 1 diabetes are often young and most of them are under 30 years old. Increasing thirst and blood sugar levels as well as frequent urination are common clinical signs [10]. In type 2, middle-aged and elderly people are more prone to the disease and are usually associated with obesity, hypertension, dyslipidemia, atherosclerosis, and other health problems. Drugs alone are not enough to treat this type of diabetes, and insulin injections are also essential [11, 12]. However, no long-term cure has been discovered for this disease, but it can be controlled with early diagnosis and prognosis in the early stages of the disease, and in the later stages of the disease, treatment can be much easier. Therefore, the prediction of diabetes has become a controversial topic for study and research [10–13].

In recent years, significant advancements have been made in developing and publishing various methods for predicting diseases, including but not limited to diabetes, Covid-19, and other illnesses [14, 15]. Simultaneously, the rapid progress of machine learning models has led to their widespread utilization in numerous applications, particularly in the medical field, for the accurate diagnosis of diverse diseases [13–16]. In general, machine learning models aim to describe and predict data and can help people make early judgments about disease based on their physical condition and diagnose the disease in its early stages until treatment is complete [14]. Amit Kishor and Chinmay Chakraborty have proposed a cutting-edge healthcare model based on machine learning techniques, aiming to enhance the accuracy and timeliness of diabetes diagnosis. In this model, they employed a set of five machine learning classifiers, including logistic regression, K-nearest neighbor, Naive Bayes, random forest, and support vector machine. Furthermore, to improve the model's performance, they utilized the fast correlation-based filter feature selection method to eliminate irrelevant features and applied the artificial minority oversampling technique to address imbalanced datasets [17]. Zou et al. [18] employed J48 decision tree, RF, and ANN models to predict diabetes from a hospital examination data set in Luzhou, China. To ensure the wide applicability of their methods, the authors selected the top-performing techniques for independent empirical tests. Chen and Pan [19] conducted a comprehensive study to predict diabetes using various machine learning methods and identify the most efficient and accurate model. The study utilized a dataset with 520 instances and 17 features, including polyuria, gender, age, sudden weight loss, polydipsia, polyphagia, weakness, irritability, genital thrush, itching, vision blurring, muscle stiffness, alopecia, and obesity. The authors compared the performance of eight classification algorithms, including Support Vector Classifier (SVC), Gaussian Naive Bayes (GNB), Random Forest (RF), Decision Tree Classifier (DTC), Logistic Regression (LR), Extra Tree Classifier (ETC), K-Nearest Neighbors (KNN), and XGBoost (XGB), and found that Extra Tree Classifier (ETC) achieved the highest accuracy of 98.55%. These results demonstrate that ETC is the most efficient and accurate machine-learning classification technique for diagnosing diabetes based on the mentioned parameters. Zhu et al. [20] proposed an innovative approach for diabetes prediction by combining PCA and K-Means techniques, resulting in a highly effective and well-clustered dataset. The model consists of three components: principal component analysis, K-Means clustering, and logistic regression, along with data standardization. The experimental outcomes demonstrated that PCA significantly enhanced the accuracy of the K-Means clustering method and the logistic regression classifier, with K-Means accurately classifying 25 data points and boosting the logistic regression accuracy by 1.98%, thereby surpassing earlier findings. Lukmanto et al. [21] utilized fuzzy support vector machines and F-exponential feature selection for the identification and classification of diabetes. Feature selection was employed to extract useful characteristics from the dataset, and the output was classified using the fuzzy inference method. The dataset was trained using SVM, resulting in an impressive accuracy of 89.02% when applied to the PIMA Indian Diabetes dataset. Furthermore, the approach employed an optimal number of fuzzy rules, maintaining an excellent level of accuracy. Raja et al. [22] proposed a novel data mining technique for type 2 diabetes prediction, combining particle swarm optimization (PSO) and fuzzy clustering (FCM) to create a highly efficient predictive model. The approach was evaluated through experiments on the PIMA Indian diabetes dataset, utilizing metrics for accuracy, sensitivity, and specificity. The results demonstrated that the proposed model outperformed other methodologies, exhibiting an 8.26% increase in accuracy compared to the other methods. Khanam et al. [23] employed seven machine learning and neural network methods to predict diabetes on the PIMA diabetes dataset. The authors created various neural network models with different numbers of hidden layers for different periods. The experimental results revealed that the models of Logistic Regression (LR) and Support Vector Machine (SVM) were highly effective in predicting diabetes and that neural networks with two hidden layers achieved an impressive accuracy of 88.6%. Rajendra et al. [24] conducted experiments on the PIMA diabetes dataset, comparing logistic regression algorithms and ensemble learning techniques for diabetes prediction. The study demonstrated that logistic regression is one of the most efficient techniques for creating prediction models and that employing feature selection, data pre-processing, and integration strategies can significantly enhance the accuracy of the model. Rawat et al. [25] conducted comparative studies on the PIMA diabetes dataset, utilizing machine learning methods such as Naive Bayesian (NB), Support Vector Machine (SVM), and Neural Network. The experimental results revealed that the neural network achieved the highest accuracy amongst all classifiers, with an impressive accuracy of 98%. The neural network method was deemed the most effective in the early detection of diabetes. Zhou et al. [26] proposed a diabetes prediction model based on Boruta feature selection and ensemble learning, which utilized unsupervised clustering of data using the K-Means +  + algorithm and stacking an ensemble learning method for classification. The model was validated on the PIMA Indian diabetes dataset, achieving an incredibly high accuracy rate of 98%, surpassing other diabetes prediction models, and highlighting its superior performance in diabetes prediction. The incorporation of Boruta feature selection and ensemble learning in this model provides a promising approach for accurate diabetes diagnosis and treatment. Shilpi et al. [27] utilized two common boosting algorithms, Adaboost.M1 and LogitBoost, to establish machine learning models for diabetes diagnosis based on clinical test data from a total of 35,669 individuals. The experimental results demonstrated that the LogitBoost classification model outperformed the Adaboost.M1 classification model, achieving an impressive overall accuracy of 95.30% with tenfold cross-validation. The authors concluded that these boosting algorithms exhibit excellent performance for diabetes classification models based on clinical medical data. The significant discriminating factors between diabetic and general populations obtained from the process of selecting preferred test items can be used as reference risk factors for diabetes mellitus. The model is also robust and has a degree of pre-diagnosis function, given that the coefficient matrix of the original data is a sparse matrix due to missing test results, some of which are directly related to disease diagnosis.

The fast-paced advancements in machine learning have shown promising results in the early detection of diseases. However, developing an accurate prediction model for diagnosing diabetes remains challenging. This is due to several factors such as the limited availability of labeled data, frequent occurrence of incomplete or missing values in the dataset, and the imbalanced nature of the dataset. These issues make it difficult to achieve optimal performance and necessitate the development of novel techniques to address them. In this article, we present an innovative pipeline-based framework for predicting diabetes in three classes of peoples (diabetic, non-diabetic, and pre-diabetic) using the Iraqi Patient Dataset for Diabetes patients (IPDD), that's an imbalanced data set. Preprocessing is an important part of the proposed framework to achieve a high-quality result. Which includes several steps, such as removing duplicate samples, filling in missing values, normalizing and standardizing data, selecting relevant features, and performing k-fold cross-validation to ensure high-quality data. Consulted with a specialist to complete missing attribute values using the k-NN Imputation method. We implemented various machine learning models, including MLMs, k-NN, SVM, DT, RF, AdaBoost, and GNB, and used Bayesian optimization and grid search techniques to find the optimal hyper-parameters for MLMs. As our dataset is imbalanced, accuracy evaluation alone is not being a good measure of model evaluation, and we therefore used the Area Under the ROC Curve (AUC) as an additional measure to evaluate the models. To evaluate the models. Under the same experimental conditions and dataset, multiple experiments are performed with different preprocessing combinations and machine learning models to maximize the area under the curve of predicting diabetes. Then optimal machine learning model is then used as the baseline model for the proposed framework to optimally predict diabetes. We then proposed an ensemble machine learning model (EMLM) with a combination of MLMs to improve the prediction accuracy and AUC of diabetes diseases. Combining different models can help address the weaknesses of using single models when the dataset is imbalanced. To combine machine learning models, we used the AUC with the One-Vs-One (OVO) multiclass classification approach as the weight for this EMLM. The AUC in the proposed EMLM is unbiased to the class distribution, and therefore, we chose it as the model's weight in the voting ensemble rather than accuracy. We performed many experiments with different combinations of MLMs to obtain the optimal set of EMLMs by applying the optimal preprocessing from previous experiments.

Feature selection and dimensionality reduction are of utmost importance in disease diagnosis research, as they enable the construction of a model with a reduced number of features. Such a model is simpler, less time-consuming for training and testing, and particularly powerful in disease prediction. In this study, we evaluated the efficacy of the MRMR feature selection method, as well as the PCA and ICA dimensionality reduction methods, in identifying the most important features affecting the target variable, i.e., the factors that have the most significant impact on the determination of class.

Our proposed framework demonstrates high accuracy and AUC in predicting diabetes, and we believe that it can be applied to other patient populations as well. By utilizing these methods, we have developed a model with fewer features, which has resulted in increased accuracy in diabetes prediction. Our study provides valuable insights into the development of a more effective diabetes prediction model, which can significantly contribute to the recovery of diabetic patients.

In conclusion, our research highlights the importance of feature selection and dimensionality reduction in disease diagnosis research, and underscores the potential of these techniques in developing accurate and efficient prediction models. Our findings can serve as a valuable resource for researchers and practitioners in the field of diabetes diagnosis and treatment, and can pave the way for future studies aimed at improving the accuracy and efficacy of diabetes prediction models.

This paper is organized as follows. "Methods" section deals with the methods used in this research, particularly the flowchart of the proposed model in section. "Machine learning models" section deals with the machine learning models, particularly the proposed EMLM in section. "Evaluation metrics" section describes the evaluation measures (metrics) of the proposed models in this article, and "Results" section describes the results of the various experiments conducted. Finally, discussions are offered in "Discussion and future work" section.

## Methods

### Dataset

**Fig. 1:**
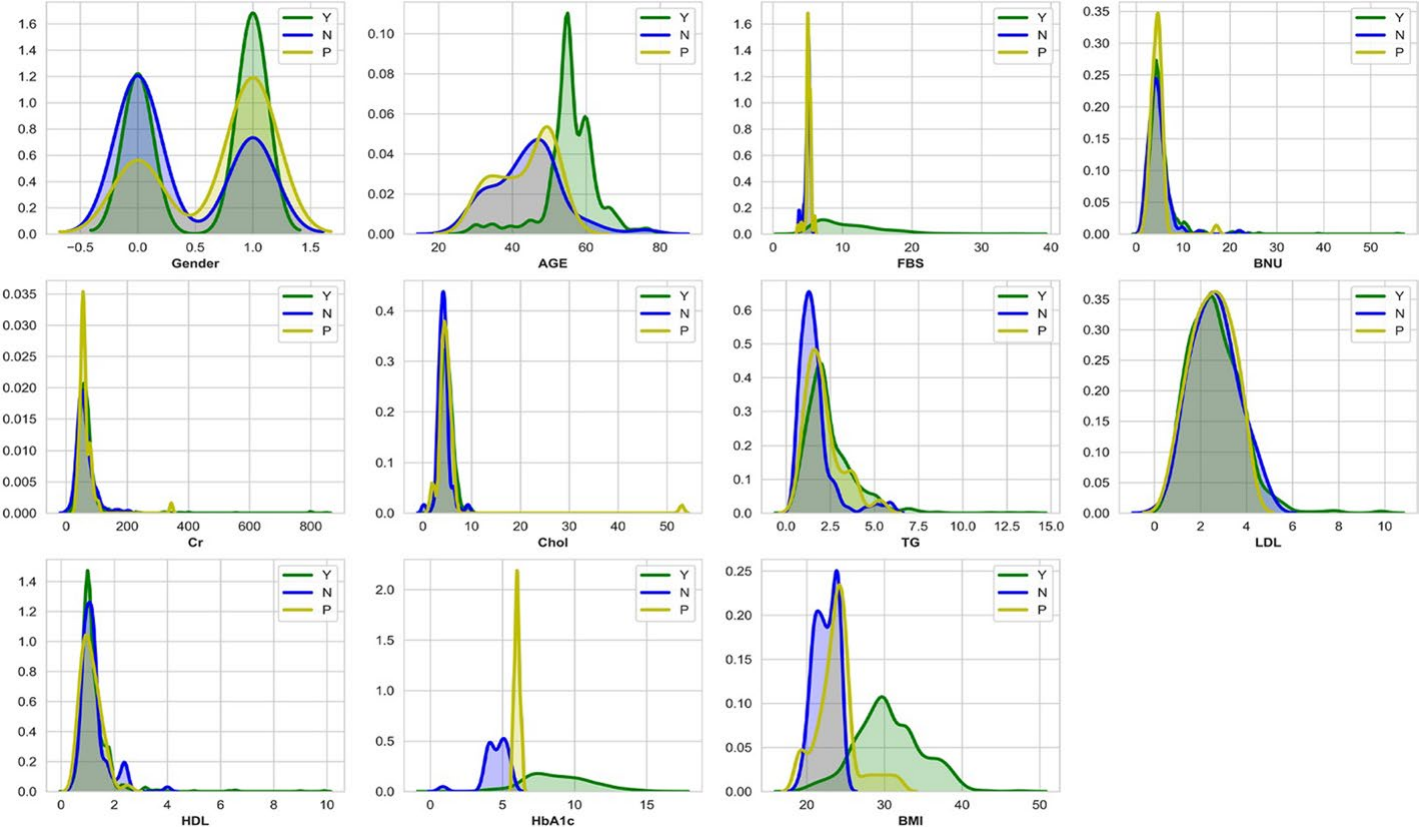
The Iraqi Patient dataset for Diabetes (IPDD) dataset population distribution of all attributes, with green, blue, and yellow color distributions denoting diabetic (Y) individuals, non-diabetic (N), and predicted diabetic (P) classes, respectively

The Iraqi Patient Dataset for Diabetes (IPDD) [28] was obtained from 1000 samples, including 565 males and 435 females aged 20–79 years old, during in-hospital physical examinations at the Specialized Center for Endocrinology and Diabetes-Al-Kindy Teaching Hospital in Iraq. This dataset is divided into three regions: Diabetic (Y) with 837 samples, Non-Diabetic (N) with 103 samples, and Predicted Diabetic (P) with 53 samples. These include 11 physical examination indicators. Table 1 lists the attribute descriptions, and the distribution of each attribute in the dataset is shown in Fig. 1, where green, blue, and yellow color distributions denote the diabetic, non-diabetic, and predicted diabetic classes, respectively.

**Table 1:** Overview of the Iraqi Patient Dataset on Diabetes (IPDD)

Attributes	Description	Mean ± Std.
Gender	0 for females and 1 for male	0.565 ± 0.4958
Age	Age in years	53.739 ± 8.8557
Fasting blood sugar (FBS)	result of a blood sample taken after a patient fasted for at least eight hours (mmol/l)	10.1443 ± 5.0844
High blood urea nitrogen (BUN)	BUN is the amount of urea nitrogen that's in your blood (mmol/l)	5.1808 ± 3.3486
Chromium (Cr)	blood levels of chromium (mmol/l)	69.28 ± 62.2764
Chol	Fast Cholesterol levels (mmol/l)	4.9092 ± 2.004
TG	Concentration Tri Glycoside Levels (mmol/l)	2.3506 ± 1.3988
LDL	Low-Density Lipoprotein (mmol/l)	2.6145 ± 1.1175
HDL	High-Density Lipoprotein (mmol/l)	1.2067 ± 0.6594
BMI	Body Mass Index (Weight in kg / (Height in m)^2^)	29.4255 ± 4.8553
Gyrated hemoglobin (HBA1C)	For the previous two to three months, average blood glucose (sugar) levels (mmol/l)	8.2623 ± 2.5370

#### Proposed framework

**Fig. 2:**
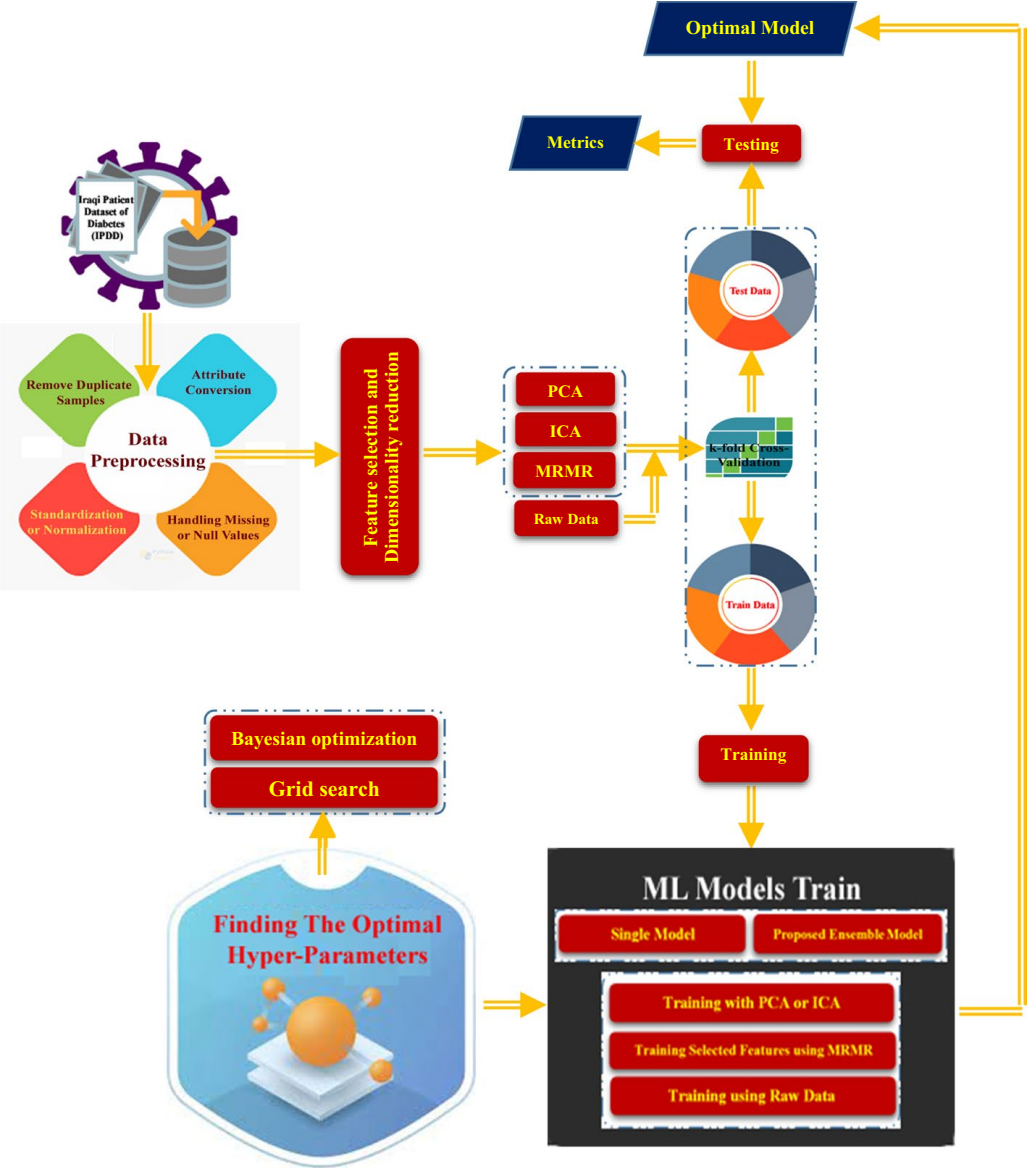
shows a potential model for reliable and automatic diabetes prediction

The framework proposed in this study is shown in Fig. 2, where the pre-processing of raw data is a crucial stage in the pipeline, as data quality can directly influence the training of the classifiers.

### Data preprocessing

**Fig. 3:**
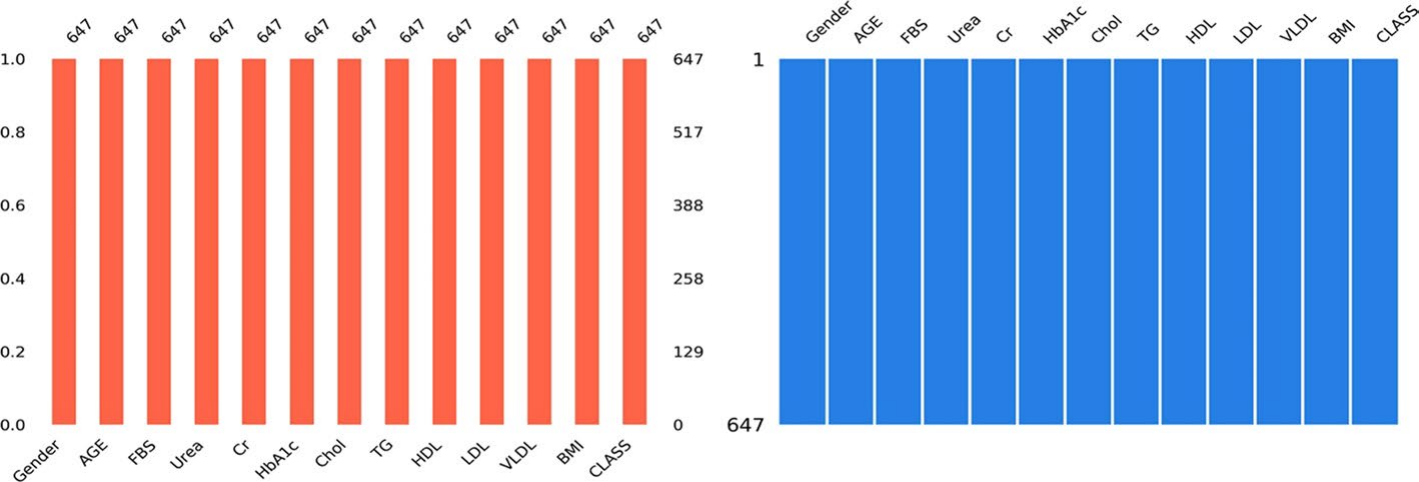
Data set after Filling in missing or null values

According to the proposed framework for predicting diabetes disease in this study (Fig. 2), data preprocessing is the first and most important step because it can improve data quality and the resulting data quality can have a direct impact on learning classification models. Preprocessing steps in the proposed framework include Removing Duplicate Samples, Converting Attributes, filling in values are missing or null (I), Normalization (N), Standardization, (Z), and attribute feature selection, and which briefly described as follows:

- *Remove Duplicate Samples* In this study, after examining all 1000 data samples, we concluded that seven of these samples were completely identical and thus were removed, leaving 993 samples.
- *Attribute Conversion* Because, in this study's data set, the values of the Gender attributes and the Class label are qualitative (non-numerical) values, for use in models, we use Eqs. (1) and (2), convert the values of these attributes to the numerical values.
  1 $$Gender \left( a \right) = \left\{ {\begin{array}{*{20}l} {0,} \hfill & {if\;a = female} \hfill \\ {1,} \hfill & {if\;a = male} \hfill \\ \end{array} } \right.$$
  2 $$Class\;Lable \left( C \right) = \left\{ {\begin{array}{*{20}l} {0,} \hfill & {if\;C = Y\;\left( {Diabetic} \right)} \hfill \\ {1,} \hfill & {if\;C = N\;\left( {Non{ - }diabetic} \right)} \hfill \\ {2,} \hfill & {if\;C = P\;\left( {Diabetic\;predicted} \right)} \hfill \\ \end{array} } \right.$$
- *Handling Missing or Null Values* Missing or null values are values that may lead to incorrect predictions or inference for each class in the classification [29, 30], so here, a little number of attribute values were completing (missing values) with the consultation of a doctor specializing in endocrinology and metabolism using the k-NN Imputation method. The result of this process is shown in Fig. 3.
- *Normalization* Data normalization is a critical factor in improving the performance of machine learning algorithms [31]. Normalization helps reduce bias from features with high numerical contributions, ensuring fair consideration of each variable during the learning process. It also enhances numerical stability, reduces training time, and facilitates meaningful feature comparisons. Because some continuous attributes in the data have a wide range of values, this can have a considerable impact on the performance of the classifier. To convert the range of continuous features to a [0,1] interval, we utilize the min–max normalization [32] as shown in Eq. (3).
  3 $$N\left( {x_{ij} } \right) = \frac{{x_{ij} - x_{jmin} }}{{x_{jmax} - x_{jmin} }}$$
  where the original attribute and the converted attribute are equal to ${x}_{ij}$ and $N\left({x}_{ij}\right)$.
- *Standardization* The Z-score [33] is a method of standardization used to convert continuous attribute numerical values into standard scores, with a mean of zero and a standard deviation of one. The Z-score standardization formula is shown in Eq. (4).
  4 $$Z\left( {x_{ij} } \right) = \frac{{x_{ij} - \overline{x}_{j} }}{{\sigma_{j} }}$$

The mean and standard deviation of the $j$th attribute is equal to ${\overline{x} }_{j}$ and ${\sigma }_{j}$ respectively.

### k-fold cross-validation

**Fig. 4:**
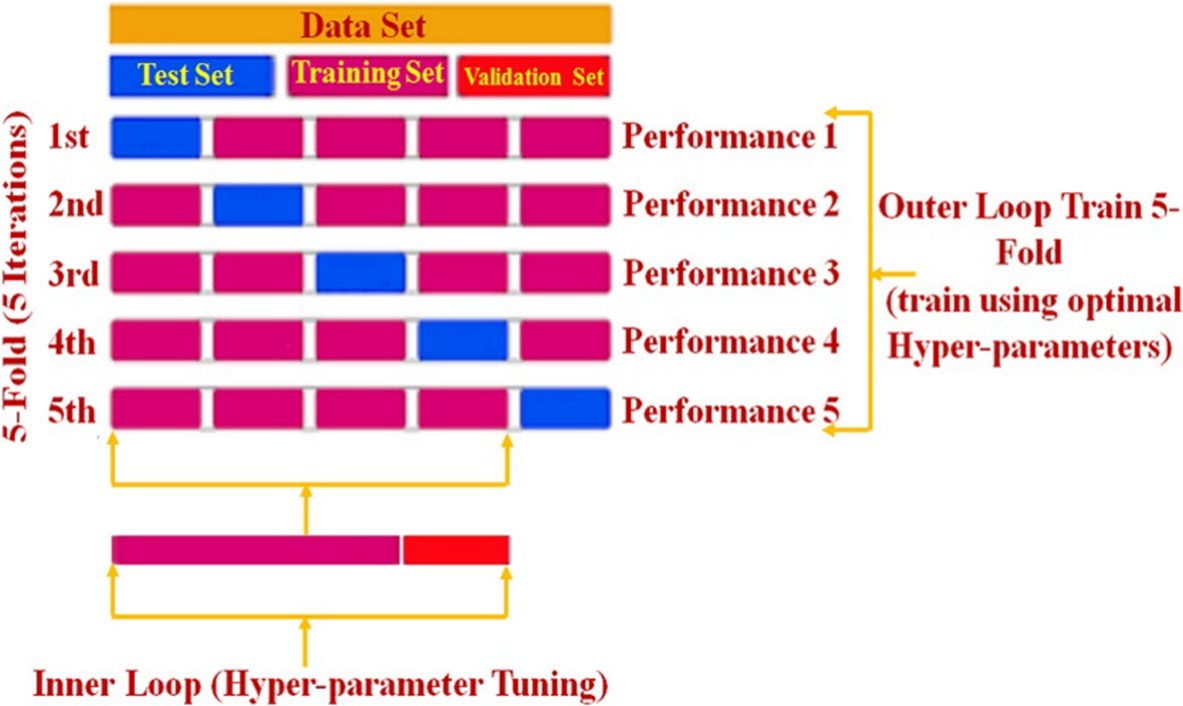
The partitioning of the IPDD dataset for kCV for both the hyper-parameters tuning and for the evaluation

K-fold Cross-Validation (kCV) is a statistical approach used to evaluate and compare the effectiveness of classifiers in machine learning algorithms. It splits data into two parts: one for training a model and the other for validation or testing [34]. In kCV, the data is separated into k equal (or nearly equal) segments or folds. Thereafter, k iterations of training and validation are performed, each iteration using a different fold of the data for validation and the remaining k-onefold for training [35, 36]. In the inner loop, where the hyper-parameter optimization algorithms (Bayesian optimization and grid search) [37–39] were applied, the hyper-parameters were trained and fine-tuned using the four folds. The test data is used to evaluate the model in the outer loop using the optimal hyper-parameters found in the training step, which are repeated five times (Fig. 4).

### Feature selection

Feature selection strategies can help reduce the number of attributes and avoid using redundant features. Various methods for feature selection and dimensionality reduction are available. To reduce dimensionality and feature selection in this study, we used PCA and ICA and Maximum Relevance Minimum Redundancy (MRMR). In this section, we describe the PCA and ICA as well as MRMR methods. In addition, the codes of these methods are included in Additional file 1: Appendix 1A.

### PCA (principal component analysis)

Principal component analysis (PCA) [40] is a mathematical approach for reducing the dimensionality of data by finding and preserving the most variation in the data set by defining directions called Principal Components. Instead of a large number of variables, each sample may be represented by a few components [18].

### ICA (independent component analysis)

The observed multivariate data, often presented as a large database of samples, is transformed into a generative model by ICA [41, 42]. The data variables in the model are viewed as linear mixtures of unknown latent variables, as is the mixture system. The independent components of the observed data are expected to be non-Gaussian and mutually independent latent variables. ICA can locate these separate components, often referred to as sources or factors. PCA and factor analysis have a superficial relationship with ICA. When these traditional methods fail, ICA is a far more effective method that can uncover the underlying causes or sources. The FastICA algorithm was used in this study [43, 44].

### MRMR (minimum redundancy maximum relevance)

Features should have the maximum Euclidean distances or have pairwise correlations as low as possible. The Minimum Redundancy Maximum Relevance (MRMR) standards, such as maximal mutual information and target phenotypes, are frequently used to supplement minimum redundancy norms. The advantages of this can be obtained in two ways. Firstly, the MRMR feature set might have a more representative target phenotype for greater generalization with the same number of features. Secondly, we can effectively cover the same space with a smaller MRMR feature set as we can with a larger regular feature set by using a smaller MRMR feature set [45–48].

## Machine learning models

### Single models

In this research, we used different classification Machine Learning Models (MLMs) such as k-NN [49, 50], multi-support vector machine Multi-Class SVM [51], DT [52], RF [53], Multi-Class AdaBoost [54–56], and GNB [57] to train and test the proposed framework. Because class boundaries may overlap, multi-classification may perform worse than binary classification. There are two methods for extending binary classification algorithms to the multi-class mode studied: The One-Vs-One (OVO) approach and the One-Vs-All (OVA) technique. However, empirical studies show that the OVO approach performs better results than the OVA approach [58]. Because our problem in this study is a multiclass classification, we used the multiclass classification OVO approach [59], all of which are pseudo-codes in Additional file 1: Appendix 1B.

After selecting the MLM, we optimized the model hyper-parameters (see Table 2) using Bayesian optimization and grid search hyper-parameter optimization methods for our desired problem.

**Table 2:** shows various MLMs with hyper-parameters that can be tuned in the internal loop using optimization approaches

MLMs	Hyper-parameters
K-NN	The number of neighbors to inspect in a k-NN **Algorithm** for computing nearest neighbors Ball Tree: A D-dimension **hyper**-parameter or ball is defined by Node KD Tree: A D-dimension point is the Leaf node Brute: based on the search using brute-force The size of the **leaf** for BT or KDT is determined by the nature of the problem The **distance** metric to use for the tree [Manhattan (${L}_{1}$- norm) or Euclidean (${L}_{2}$- norm)]
SVM	The type of kernel function (Linear, Polynomial, RBF, sigmoid) ***C***: Penalty parameter (The ***C*** parameter controls how much you want to punish your model for each misclassified point for a given curve) Gama: Kernel coefficient (Gamma parameter in Radial basis function, polynomial, and sigmoid kernels, controls the distance of influence of a single training point) **Decision**_function_shape or multi-classification approach (OVA or OVO)
DT	Criterion function: Gini (Gini impurity) or entropy (information gain) The method for selecting the split at each node The tree's maximum depth The bare minimum of samples is needed to split an internal node The bare minimum of samples is required at each leaf node. The total weights' minimum weighted fraction The number of features to take into account when looking for the ideal split
RF	The N of Decision Trees in the forest The Criteria which to split on at each node of the trees: (Gini or Entropy for classification) The maximum depth of the individual trees At an internal node, a minimal number of samples to divide on. Maximum number of leaf nodes Number of random features The size of the bootstrapped dataset
AdaBoost	The boosting algorithm Real boosting Discrete boosting Learning rate to shrink the contribution of each classifier The maximum number of estimators to terminate the boosting
GNB	Variance smoothing (the portion of the largest variance of all features)

### The proposed ensemble of machine learning models

Within the communities of computational intelligence and machine learning, multiple classifier systems, often known as ensemble systems, have received much press. In a wide range of problem areas and real-world applications, ensemble systems have proven to be incredibly successful and versatile; thus this focus is well-deserved [60–62]. The Ensemble of the Machine Learning Model (EMLM) is a well-known approach for improving performance by grouping a set of classifiers (especially here where the data is imbalanced) [63, 64]. The aggregation of the output from various models can improve the precision of the prediction [39]. For an ensemble of MLMs, the output of each MLM is a function ${Y=f}_{j}: X\to {R}^{C}$ that assigns $C$ confidence values ${P}_{i}\in {\mathbb{R}}$ to an unseen test sample $X$, where ${P}_{i}^{e}\in \left[\mathrm{0,1}\right]$ for $i=\mathrm{1,2},\dots , C$, and ${\sum }_{i=1}^{C}{P}_{i}=1$, and $j=\mathrm{1,2},3,\dots ,m$ is the number of MLMs. (In our case, the values ${P}_{1}, {P}_{2}, \mathrm{and} {P}_{3}$ indicate confidence values for each MLM). In this study, we utilized a weighted aggregation model in which the sum of individual confidences is calculated as:

5 $$P_{i}^{EMLM} = \frac{{\mathop \sum \nolimits_{j = 1}^{5} \left( {A_{j} \times P_{ij} } \right)}}{{\mathop \sum \nolimits_{i = 1}^{3} \mathop \sum \nolimits_{j = 1}^{5} \left( {A_{j} \times P_{ij} } \right)}}, \;\;i = 1,2,3$$

where ${P}_{ij}$ denotes the ${MLM}_{j}$ confidence that $X$ (an unseen test sample) belongs to class ${\mathrm{c}}_{\mathrm{i}}$ and ${\mathrm{A}}_{\mathrm{j}}$ is the weight corresponding to the OVO approach AUC of that *j*th $\mathrm{MLM}$. The normalization term is used to convert values ${P}_{i}^{EMLM}$ to a $\left[\mathrm{0,1}\right]$ interval for $i=\mathrm{1,2},3$ such that ${\sum }_{i=1}^{3}{P}_{i}^{EMLM}=1$. The ensemble model's output, $Y\in {\mathbb{R}}^{C}$, has ${P}_{i}^{EMLM}$ confidence values. Finally, the unseen test sample $X$ belongs to the class with the maximal probability; That is, ${C}_{i}$, if ${P}_{i}^{EMLM}=max(Y=f(X))$.

## Evaluation metrics

In this study, we used various metrics for evaluating the Multiclass Classification Model Evaluation to measure the performance of the MLMs [65, 66], as shown in Table 3.

**Table 3:** The summary of all performance evaluation measure metrics of Multiclass classification models

Measures	Definitions	Formula
Average accuracy	The classifier's average per-class effectiveness	$\frac{{\sum }_{i=1}^{k}\frac{{tp}_{i}+{tn}_{i}}{{tp}_{i}+{tn}_{i}+{fp}_{i}+{fn}_{i}}}{k}$(8)
Micro-averaging		
Precision	The genuine class labels' average per-class agreement with the classifier's labels	$\frac{\sum_{i=1}^{k}{tp}_{i}}{\sum_{i=1}^{k}{tp}_{i}+{fp}_{i}}$(9)
Recall	A classifier's average per-class efficacy in identifying class labels	$\frac{\sum_{i=1}^{k}{tp}_{i}}{\sum_{i=1}^{k}\left({tp}_{i}+{fn}_{i}\right)}$(10)
F1-score	The macro-average precision and recall's harmonic mean	$\frac{2\times Precision\times Recall}{Precision+Recall}$(11)
ROC (AUC)	Receiver operating characteristics (ROC) with the area under the ROC curve (AUC also measured the ranking of predictions rather than their absolute values)	

$TN$ stands for True Negative, and it refers to the number of cases that have been correctly diagnosed as negative. $TP$ stands for True Positive, and it refers to the number of positive examples that have been correctly detected. The letters $FP$ stand for False Positive, which refers to the number of real negative cases classified as positive; $FN$ stands for False Negative, which refers to the number of genuine positive examples classified as negative; and *k* stands for the total number of classes. Rather than giving absolute results, we used Receiver Operating Characteristics (ROC) and the Area Under the ROC Curve (AUC) to determine how well predictions are assessed.

## Results

Table 4 illustrates the quantitative results for choosing the most effective preprocessing and machine learning model, with the average accuracy (AAC) and standard deviation presented for comparison. Each model's ability to achieve the optimal AAC using the proposed pipeline is summarized in Table 4, along with the effective preprocessing and feature selection technique, as well as the number of attributes chosen. The optimally tuned hyper-parameters using hyper-parameter optimization methods are also shown in Table 4.

**Table 4:** illustrates the optimal-performing MLM and preprocessing, as well as tuned hyper-parameters with the highest AAC

MLMs	Optimal-performing preprocessing	Hyper-parameter tuning methods	Optimal hyper-parameters	Performance
k-NN	I + N MRMR = 6	Grid search	Algorithm = auto leaf_size = 5 n_neighbors = 25 weight = uniform ${L}_{2}$- norm (Euclidean)	$0.971\pm 0.003$
SVM	I + N	Bayesian optimization	C = 1 Gamma = 0.1 Kernel = RBF Decision_function_shape = OVO	$0.948\pm 0.003$
DT	I + Z MRMR = 10	Bayesian optimization	Criterion = gini bootstrap = True min_samples_leaf = 1 max_depth = 8 max_features = auto min_samples_leaf = 2 min_samples_split = 0.2	$0.968\pm 0.003$
RF	I + N MRMR = 6,8,10	Bayesian optimization	Criterion = gini n_estimator = 150 bootstrap = True min_samples_leaf = 1 max_depth = 8 max_features = sqrt	$0.988\pm 0.003$
GNB	I + Z + PCA = 12	Grid search	var_smoothing = 08112	$0.926\pm 0.006$
AdaBoost	I + MMR = 10	Grid search	boosting algorithm = AdaBoost.MH n_estimator = 150 learninh_rate = 0.1	$0.961\pm 0.003$

Tables 5, 6, 7 and 8 show that using appropriate preprocessing can improve the outcomes of various models.

**Table 5:** Results of the first experiment in terms of AAC for MLMs, using all features in different modes of pre-processing combination

Preprocessing	N	k-NN	SVM	DT	RF	GNB	AdaBoost	Best MLM
I	11	$0.928\pm 0.0$ 00	$0.960\pm 0.0$ 01	$0.964\pm 0.001$	$0.974\pm 0.001$	$0.920\pm 0.000$	$0.961\pm 0.001$	RF
I + N	11	$0.962\pm 0.004$	$0.964\pm 0.0$ 03	$0.929\pm 0.004$	$0.975\pm 0.002$	$0.957\pm 0.001$	$0.960\pm 0.002$	RF
I + Z	11	$0.960\pm 0.002$	$0.968\pm 0.0$ 02	$0.964\pm 0.003$	$0.978\pm 0.003$	$0.958\pm 0.002$	$0.962\pm 0.002$	RF

**Table 6:** Results of the second experiment in terms of AAC for MLMs, using the MRMR feature selection algorithm in different modes of preprocessing combination

Preprocessing	Algorithm	N	k-NN	SVM	DT	RF	GNB	AdaBoost	Best MLM
I	MRMR	4	$0.931\pm 0.001$	$0.920\pm 0.001$	$0.916\pm 0.001$	$0.981\pm 0.001$	$0.922\pm 0.001$	$0.940\pm 0.001$	RF
	MRMR	6	$0.941\pm 0.002$	$0.924\pm 0.002$	$0.963\pm 0.002$	$0.986\pm 0.00$ 2	$0.936\pm 0.002$	$0.950\pm 0.002$	RF
	MRMR	8	$0.930\pm 0.002$	$0.928\pm 0.002$	$0.950\pm 0.002$	$0.986\pm 0.00$ 2	$0.940\pm 0.002$	$0.954\pm 0.002$	RF
	MRMR	10	$0.914\pm 0.002$	$0.932\pm 0.002$	$0.962\pm 0.002$	$0.985\pm 0.00$ 2	$0.946\pm 0.002$	$0.961\pm 0.002$	RF
I + N	MRMR	4	$0.969\pm 0.001$	$0.940\pm 0.002$	$0.944\pm 0.002$	$0.981\pm 0.00$ 2	$0.948\pm 0.002$	$0.940\pm 0.002$	RF
	MRMR	6	$0.958\pm 0.002$	$0.944\pm 0.002$	$0.935\pm 0.002$	$0.985\pm 0.00$ 2	$0.950\pm 0.002$	$0.952\pm 0.002$	RF
	MRMR	8	$0.964\pm 0.003$	$0.931\pm 0.003$	$0.931\pm 0.003$	$0.986\pm 0.00$ 3	$0.952\pm 0.003$	$0.944\pm 0.003$	RF
	MRMR	10	$0.971\pm 0.003$	$0.948\pm 0.003$	$0.958\pm 0.003$	$0.988\pm 0.00$ 3	$0.956\pm 0.003$	$0.946\pm 0.003$	RF
I + Z	MRMR	4	$0.931\pm 0.001$	$0.913\pm 0.001$	$0.940\pm 0.001$	$0.972\pm 0.00$ 1	$0.960\pm 0.001$	$0.942\pm 0.001$	RF
	MRMR	6	$0.930\pm 0.002$	$0.920\pm 0.002$	$0.947\pm 0.002$	$0.984\pm 0.00$ 2	$0.962\pm 0.001$	$0.940\pm 0.00$ 2	RF
	MRMR	8	$0.930\pm 0.002$	$0.930\pm 0.002$	$0.948\pm 0.002$	$0.976\pm 0.00$ 2	$0.964\pm 0.003$	$0.943\pm 0.003$	RF
	MRMR	10	$0.924\pm 0.003$	$0.916\pm 0.003$	$0.968\pm 0.003$	$0.977\pm 0.002$	$0.967\pm 0.003$	$0.945\pm 0.003$	RF

**Table 7:** The results of the second experiment in terms of AAC for MLMs, using the PCA dimensionality reduction algorithm in different cases of pre-processing combination

Preprocessing	Algorithm	N	k-NN	SVM	DT	RF	GNB	AdaBoost	Best MLM
I + N	PCA	10	$0.754\pm 0.030$	$0.900\pm 0.001$	$0.934\pm 0.001$	$0.968\pm 0.003$	$0.962\pm 0.002$	$0.854\pm 0.001$	RF
	PCA	$11$	$0.757\pm 0.034$	$0.902\pm 0.002$	$0.953\pm 0.002$	$0.960\pm 0.003$	$0.966\pm 0.004$	$0.900\pm 0.002$	RF
	PCA	12	$0.817\pm 0.008$	$0.908\pm 0.003$	$0.952\pm 0.001$	$0.964\pm 0.003$	$0.971\pm 0.001$	$0.914\pm 0.003$	GNB
I + Z	PCA	10	$0.826\pm 0.004$	$0.910\pm 0.005$	$0.820\pm 0.003$	$0.962\pm 0.003$	$0.974\pm 0.002$	$0.918\pm 0.001$	GNB
	PCA	11	$0.829\pm 0.001$	$0.914\pm 0.004$	$0.867\pm 0.004$	$0.970\pm 0.002$	$0.972\pm 0.002$	$0.920\pm 0.005$	GNB
	PCA	12	$0.830\pm 0.0$ 20	$0.919\pm 0.006$	$0.901\pm 0.005$	$0.964\pm 0.001$	$0.977\pm 0.001$	$0.926\pm 0.006$	GNB

**Table 8:** The results of the second experiment in terms of AAC for MLMs, using the ICA algorithm in different cases of pre-processing combination

Preprocessing	Algorithm	N	k-NN	SVM	DT	RF	GNB	AdaBoost	Optimal MLM
I + N	ICA	4	$0.826\pm 0.011$	$0.890\pm 0.010$	$0.950\pm 0.004$	$0.951\pm 0.010$	$0.951\pm 0.003$	$0.930\pm 0.002$	RF
	ICA	$5$	$0.867\pm 0.012$	$0.900\pm 0.010$	$0.956\pm 0.005$	$0.958\pm 0.010$	$0.957\pm 0.002$	$0.931\pm 0.003$	GNB
I + Z	ICA	4	$0.880\pm 0.014$	$0.908\pm 0.004$	$0.959\pm 0.002$	$0.956\pm 0.005$	$0.962\pm 0.001$	$0.938\pm 0.004$	GNB
	ICA	5	$0.892\pm 0.011$	$0.910\pm 0.004$	$0.961\pm 0.006$	$0.961\pm 0.003$	$0.966\pm 0.003$	$0.941\pm 0.003$	GNB

As one can see, we used four different and integrated experiments to compare classification MLMs (Tables 5, 6, 7 and 8). When the eleven features are used, all classifiers show optimal performance for missing values filling (I) and standardization (Z) (Table 5). According to Table 5, the first experiment shows that the RF classifier outperforms other classifiers when using all features with different combinations of data pre-processing. With I + N pre-processing, the k-NN classifier exhibits the highest AAC compared with other pre-processing methods for this classifier. Similarly, the SVM, DT, GNB, and AB classifiers achieve the highest AAC using I + Z pre-processing, surpassing other pre-processing techniques.

In the second experiment (Table 6) and using MRMR (with 10 features) to select the features of the k-NN classifier in the I + N pre-processing case, it had the highest AAC compared to other pre-processing's for this classifier. In this experiment, the SVM classifier also had the highest AAC compared to other pre-processing's and feature selection by MRMR for this algorithm by selecting 10 features by MRMR and using I + N. The DT, GNB, and AB classifiers also respectively had the highest AAC value with I + Z, I + Z, and I pre-processing and the number of features equal to 10, 10, and 8 in this experiment compared to other pre-processing's and the number of features selected by MRMR. However, for the RF classifier, in this experiment, the use of I + N pre-processing 10 features obtained the highest AAC value compared to all the different states of this experiment and was chosen as the optimal classifier in this experiment.

According to Table 7, as you can see, using PCA for dimensionality reduction in three cases with explained variance equal to 90%, 95%, and 98%, and with all different cases of pre-processing did not improve the AAC value compared to other experiments. Thus, in this data, it is not appropriate to use PCA to reduce the dimension (in the third experiment).

Finally, in the fourth experiment, using ICA with five components and I + Z pre-processing, different classifiers were optimized in terms of performance, and AAC values were more optimized than in other experiments. Among all the classifiers, GNB could perform optimally in terms of AAC performance (Table 8). Because GNB is sensitive to the distribution shape and assumes that data is derived from a normal distribution, we observe in all experiments that, after outlier standardization, the performance of AAC of this classification also increases (Tables 5, 6, 7 and 8).

**Fig. 5:**
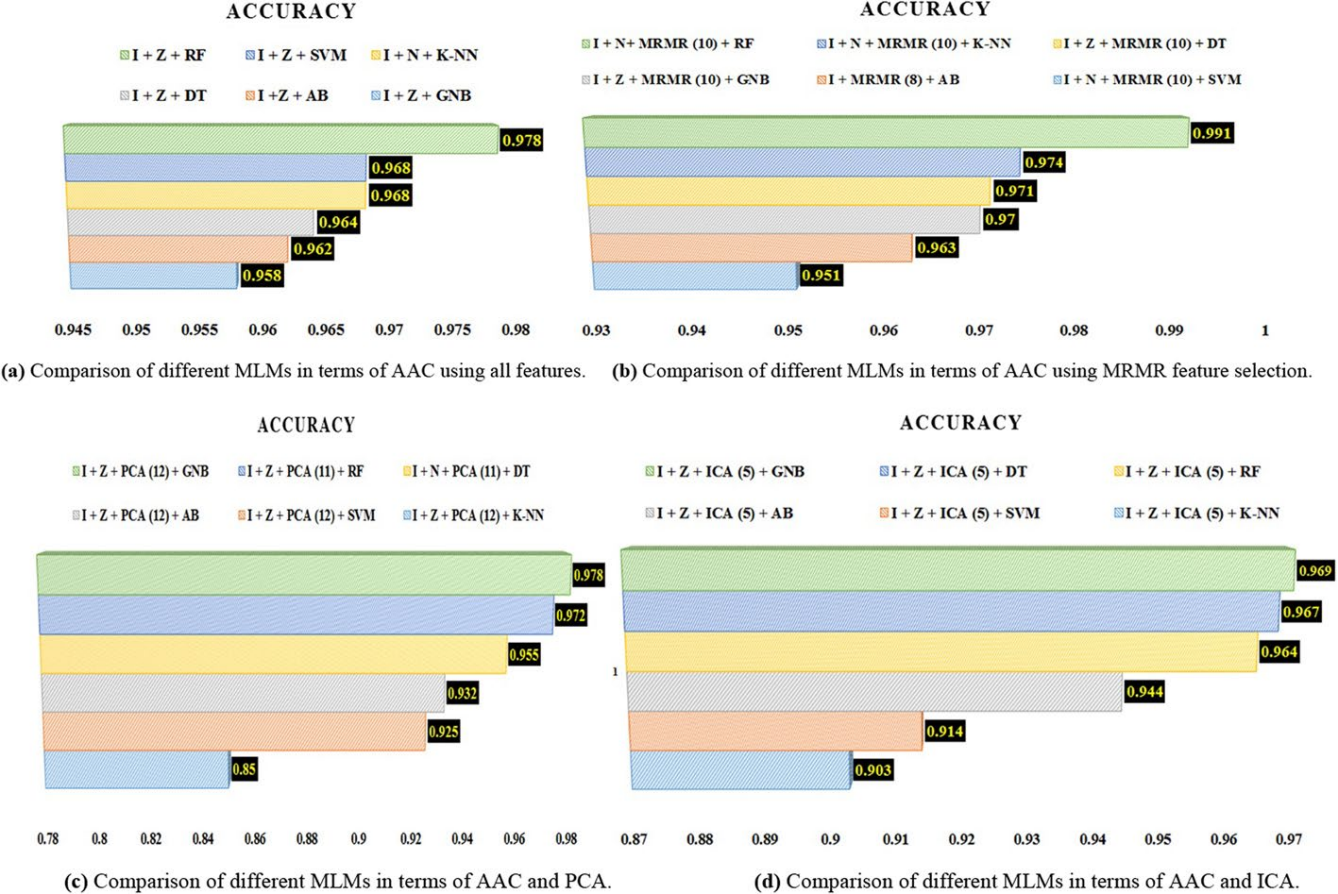
Comparison of different MLMs in terms of AAC in 4 experiments. **a** Comparison of different MLMs in terms of AAC using all features. **b** Comparison of different MLMs in terms of AAC using MRMR feature selection. **c** Comparison of different MLMs in terms of AAC and PCA. **d** Comparison of different MLMs in terms of AAC and ICA

Figure 5 visually compares the optimal results of each of the four experiments. Figure 5a shows that, in the first experiment with 11 features, the RF classifier with I + Z pre-processing performed more successfully than in other cases. Part b of Fig. 5 also shows that, in the second experiment, the RF classifier and the selection of 6, 8, and 10 features using MRMR and I + N pre-processing performed better in terms of AAC than in other cases. Part c also shows the results of the third experiment, which used PCA to reduce the dimension, that the GNB classifier with PCA, a variance value of 98%, and I + Z pre-processing provided better performance than other cases. Finally, part d also shows the results of using ICA, and in this case, the GNB classifier with ICA, a number of components of 5, and pre-processing I + Z had the highest AAC value compared to other cases.

**Fig. 6:**
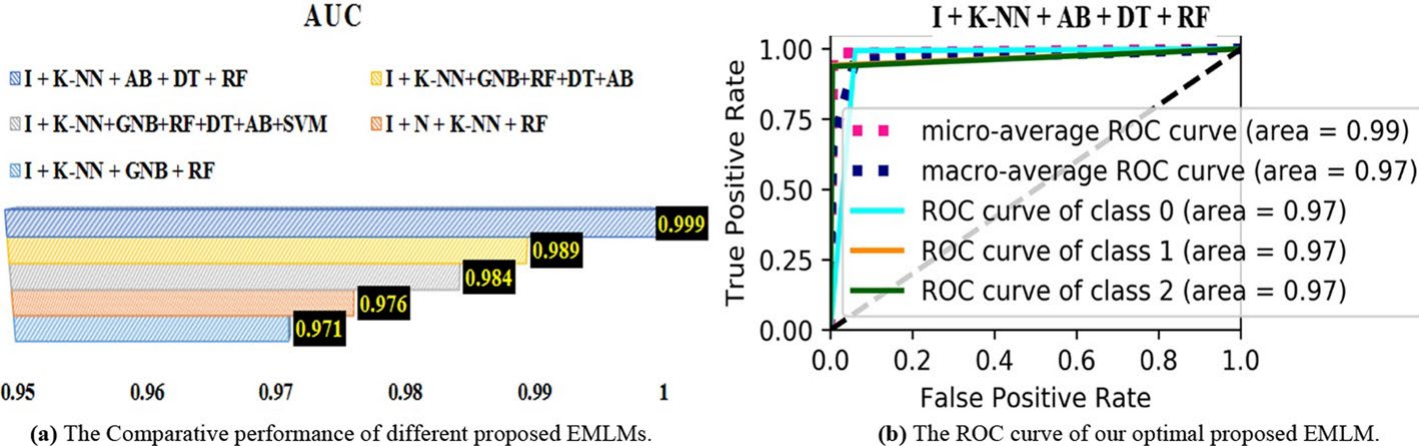
The Comparative performance of different proposed **a** MLMs, and **b** the ROC curve of our optimal proposed EMLM. **a** The Comparative performance of different proposed EMLMs. **b** The ROC curve of our optimal proposed EMLM

Optimal pre-processing from Table 4 is used in this experiment. The combination of the six MLMs provides several EMLMs. Table 9 shows the optimal performing EMLM with two, three, four, five, and six baseline models, as well as their outcomes. As shown in Table 9, the combination of K-NN, AB, DT, RF, and I pre-processing yields the highest results for diabetes prediction across all performance evaluation measures. The AUC bar chart of the optimal EMLMs is shown in Fig. 6a. The curve of Fig. 6b shows the optimal EMLM model in terms of AUC.

**Table 9:** Comparative performance of ensemble machine learning models on various micro-averaging measures

EMLMs	Precision	Recall	F1-Score	Accuracy	AUC
I + N + K-NN + RF	$0.910\pm 0.002$	$0.857\pm 0.002$	$0.877\pm 0.002$	$0.996\pm 0.0001$	$0.974\pm 0.002$
I + K-NN + GNB + RF	$0.903\pm 0.002$	$0.870\pm 0.00$ 5	$0.870\pm 0.410$	$0.998\pm 0.0000$	$0.965\pm 0.006$
I + K-NN + AB + DT + RF	$0.986\pm 0.001$	$0.979\pm 0.002$	$0.985\pm 0.001$	$0.998\pm 0.0007$	$0.999\pm 0.000$
I + K-NN + GNB + RF + DT + AB	$0.940\pm 0.002$	$0.873\pm 0.006$	$0.897\pm 0.010$	$0.998\pm 0.0003$	$0.988\pm 0.001$
I + K-NN + GNB + RF + DT + AB + SVM	$0.940\pm 0.002$	$0.910\pm 0.001$	$0.903\pm 0.160$	$0.998\pm 0.0003$	$0.980\pm 0.004$

**Fig. 7:**
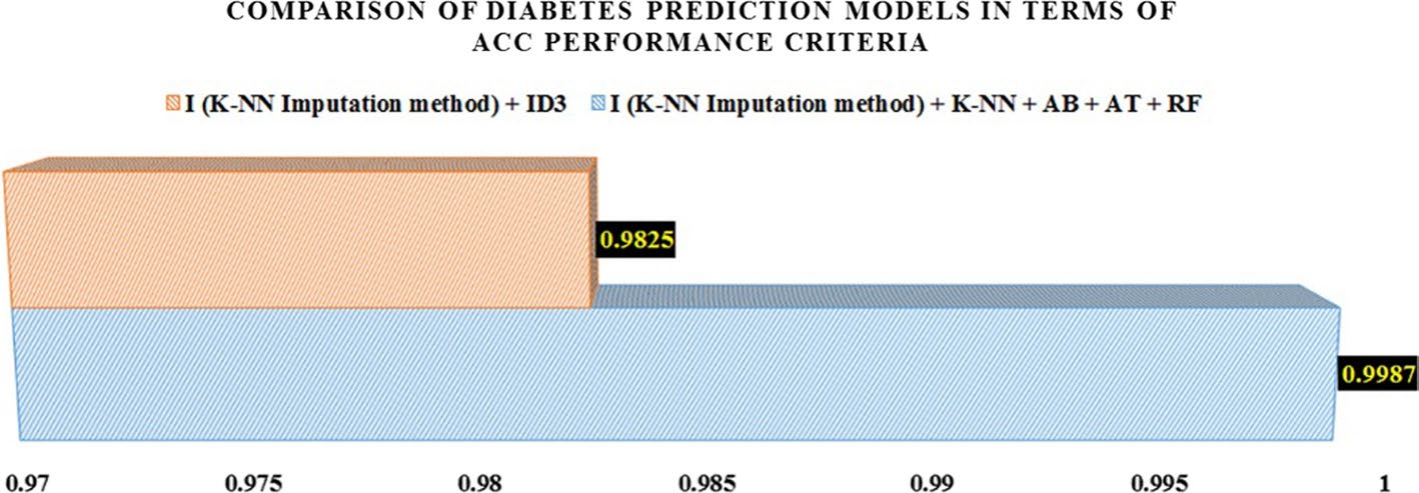
Comparison of diabetes prediction models in terms of ACC performance criteria

Finally, as shown in Table 10, the combination of K-NN, AB, DT, and RF gives the highest results for predicting diabetes in all performance evaluation criteria compared to the only models presented on the PIDD dataset (the Hybrid model presented by Soukaena Hassan et al. [67]). Additionally, the bar charts shown in Fig. 7 graphically compare these two models with each other in terms of accuracy evaluation criteria.

**Table 10:** Comparison of diabetes prediction models in terms of ACC performance criteria

Researchers	Proposed model	ACC (%)
Soukaena Hassan et al. [67]	Designing a diabetes Hybrid diagnosis system by combining KNN, and ID3 algorithms (I)	98.25
Current study	Combination of K-NN, AB, DT, and RF classification models (I)	99.87

## Discussion and future work

Diabetes is a chronic condition that significantly impacts individuals' quality of life, underscoring the critical need for accurate prediction methods in its management and prevention. In our study, we delve into the analysis and interpretation of results obtained from our ensemble machine learning models, which were designed to predict diabetes using the IPDD dataset. We also explore the implications of our findings, discuss the limitations of our study, and provide recommendations for future research.

The primary contribution of this research lies in introducing a introduces, pipeline-based framework of multi-class machine learning models for diabetes prediction. The framework utilizes the IPDD dataset, which encompasses three distinct groups: diabetic subjects (Y), non-diabetic subjects (N), and predicted diabetic subjects (P). The innovative nature of this framework lies in its ability to effectively classify individuals into these categories, thereby enhancing our understanding of diabetes prediction. This approach addresses the multi-class classification problem and ensures a comprehensive evaluation of performance by employing various evaluation metrics to assess the effectiveness of our proposed models. Data pre-processing plays a vital role in enhancing the accuracy and efficiency of predictive models. In our proposed model, we utilized several pre-processing techniques, such as filling missing values, standardization, normalization, feature selection, and dimensionality reduction. These techniques were implemented to meticulously prepare the data, improve model performance, and mitigate the impact of incomplete or inconsistent data. The results of our study emphasize the significance of data pre-processing in achieving accurate predictions for diabetes. By leveraging the collective intelligence of multiple individual classifiers, our ensemble approach demonstrates its effectiveness through improved overall performance and accuracy in diabetes prediction. This approach addresses biases and errors inherent in individual classifiers, which is particularly important given the challenges posed by imbalanced data and missing attribute values in diabetes prediction. Our experiments consistently showed that the random forest model, in combination with the MRMR and I + N stages of data pre-processing, outperformed other models. This highlights the importance of feature selection and dimensionality reduction techniques in enhancing diabetes prediction accuracy. The utilization of MRMR feature selection and PCA/ICA dimensionality reduction methods enables the identification of key features that significantly impact class determination. Furthermore, combining K-NN, AB, DT, and RF models with 11 features and I pre-processing exhibited superior performance in predicting diabetes in the IPDD dataset. This underscores the significance of employing a diverse set of machine learning models in an ensemble approach to enhance prediction accuracy. By harnessing the strengths of these models, we achieve more robust and reliable predictions. It is important to note that the evaluation of our models was not solely based on accuracy due to the imbalanced nature of the dataset. Instead, we employed multiple evaluation measures, including the Area Under the ROC Curve (AUC), to provide a comprehensive assessment of model performance. AUC is particularly suitable for imbalanced datasets as it considers the trade-off between true positive rate and false positive rate, offering a more accurate representation of the model's predictive power. Despite yielding promising results, our study has certain limitations. Firstly, the IPDD dataset used in our research may possess inherent biases and limitations that could affect the generalizability of our findings to other populations. Future studies should consider incorporating datasets from diverse patient populations to validate the effectiveness of our proposed models. Secondly, while we employed various data pre-processing techniques, there may be alternative approaches that could further optimize the performance of our models. Exploring alternative pre-processing techniques and comparing their efficacy could be a valuable avenue for future research. Ensemble models have their limitations, including increased model complexity, longer training and testing times, and the requirement of comprehensive data for model construction and configuration. Additionally, the interpretation of results from these models can be challenging due to their complexity across different datasets, potentially leading to inconclusive outcomes. Therefore, prior to utilizing these models, a meticulous examination and in-depth analysis of their features, data size, and other aspects are imperative.

In conclusion, our research demonstrates the potential of ensemble machine learning models, along with comprehensive data pre-processing techniques, in accurately predicting diabetes using the IPDD dataset. The results highlight the importance of feature selection and dimensionality reduction in improving prediction accuracy. Our proposed models offer a promising approach to diabetes prediction by addressing challenges posed by imbalanced data and missing attribute values. The findings of this study contribute to the field of diabetes diagnosis and treatment, providing valuable insights for researchers and practitioners. Our future research will focus on validating our models with larger and more diverse datasets, investigating additional preprocessing techniques to enhance the performance of diabetes prediction models, as well as exploring novel methods for early detection of COVID-19 disease and applications in mobile computing and manufacturing for comprehensive early disease diagnosis.

### Supplementary Information

**Additional file 1.** Appendix for diabetes disease predication, (a) algorithms for reduce dimensionality and feature selection and (b) MLMs.

## Data Availability

Data were used from a publicly available dataset [28] (https://data.mendeley.com/datasets/wj9rwkp9c2/1) (Note: Of course, it should be noted that the data set in this link does not have the attribute value FBS. Through correspondence with the person responsible for this dataset [67] we obtained the values of this feature and added it to the dataset).
