# Supplementary material for: Prediction of diabetes disease using an ensemble of machine learning multi-classifier models
Source: BMC Bioinformatics. 2023 Sep 12;24:337. doi: 10.1186/s12859-023-05465-z (PMC10496262; doi:10.1186/s12859-023-05465-z)
Supplement: Supplementary file 1 — Additional file 1. Appendix for diabetes disease predication, (a) algorithms for reduce dimensionality and feature selection and (b) MLMs. [file 12859_2023_5465_MOESM1_ESM.docx]

**Appendix for diabetes disease predication, (a) algorithms for reduce** **dimensionality and feature selection and (b) MLMs**

1. **algorithms for feature selection**

| **FastICA-based Feature Selection Algorithm 1: Implementation Steps** |
| --- |
| **Input**: $n$-dimensional original data, ${X\mathcal{\in R}}^{n}$ with $N$ samples.  **Output:** $W$ de-mixing matrix  1 Calculate the mean $\bar{X}\in\mathcal{R}^{n}$ of ${X\mathcal{\in R}}^{n}$, $\bar{X}=\frac{1}{N}\sum_{i=1}^{N} X_{i}$ To make the observed data set zero mean, the mean is subtracted from it. $X_{c}=X-\bar{X}$.  2 Whitening. The centered data $X_{c}$'s covariance matrix $Cov(X)$ is computed. $Cov(X)$ eigenvalue decomposition is conducted. $Z= \frac{E\times X_{c}}{\sqrt{D}}$ if $D$ is the eigenvalue  matrix and $E$ is the eigenvector matrix.  3 For one unit, there are three fixed-point iterations. One row of the de-mixing matrix $W$ is estimated as a vector $W^{T}$ that is an extremum of contrast functions  by the rapid ICA technique for one unit. Estimation of W is done iteratively, utilizing the processes listed below, until convergence is attained.  3.1 Choose a unit norm random vector$W$ as the starting point.  3.2 $W_{p}=\bar{(Zg(W_{p}^{T}Z))}-\bar{(Z\acute{g}(W_{p}^{T}Z)})W$, where  $\left\{ \begin{aligned} g_{1}\left( W_{p}^{T}z \right)=\tanh\left( aW_{p}^{T}z \right) \\ \acute{g_{1}}\left( W_{p}^{T}z \right)=a\left( 1-{tanh}^{2} \left( aW_{p}^{T}z \right) \right) 1\leq a\leq2 \end{aligned} \right.$  $\left\{ \begin{aligned} g_{2}\left( W_{p}^{T}z \right)=\exp\left( -\frac{\left( W_{p}^{T}z \right)^{2}}{2} \right) \\ \acute{g_{2}}\left( W_{p}^{T}z \right)=\left( 1-{(W_{p}^{T}z)}^{2} \right)\exp\left( -\frac{\left( W_{p}^{T}z \right)^{2}}{2} \right) \end{aligned} \right.$  $\left\{ \begin{aligned} g_{3}\left( W_{p}^{T}z \right)={(W_{p}^{T}z)}^{3} \\ \acute{g_{3}}\left( W_{p}^{T}z \right)={3(W_{p}^{T}z)}^{2} \end{aligned} \right.$  3.3 $W_{p}^{*}=\frac{W_{p}}{\vert\vert W_{p}\vert\vert}$ where $\vert\vert W_{p}\vert\vert$ is the norm of $W$  3.4 If $\vert W\left( old \right)-W(new)\vert\leq\varepsilon$ is not fulfilled, return to Step $3$, where $\varepsilon$ is a convergence parameter$({\sim10}^{-4})$ and $W\left( old \right)$ is the previous value of $W$  before it is replaced by the newly calculated Value $W(new)$  4 The second independent component is assessed. To estimate the other ICs, repeat steps 3 of the procedure to obtain weight vectors $W_{i}$ $i=1,2,3,\ldots,n.$  The weight vectors are decorrelated using Gram–Schmidt style orthogonalization to prevent distinct vectors from converging to the same optimum and  thus the same IC. When the p vectors $W_{i}$ $i=1,2,3,\ldots,p$ have been estimated, Step $3$ is executed for $W_{p+1}$, and the following iteration steps are conducted  after each iteration step.  $W_{p+1}= W_{p+1}-\sum_{j=1}^{p} (W_{p+1}^{T}W_{j})W_{j}$  $W_{p+1}=\frac{W_{p+1}}{\sqrt{W_{p+1}^{T}W_{p+1}}}$ |

| **PCA-based Feature Selection Algorithm 1: Implementation Steps** |
| --- |
| **Part 1:**  **Input**: $n$-dimensional original data, ${X\mathcal{\in R}}^{n}$ with $N$ samples.  **Output:** $Eigenvectors V and eigenvalues \lambda$  1 Subtract the mean from each feature:  X_centered = X - mean($X$)  2 Calculate the covariance matrix:  C = cov (X_centered)  3 Calculate the eigenvectors and eigenvalues of C:  V, λ = eig (C)  **Part 2:**  **Input:** Eigenvectors V and eigenvalues λ, original data matrix X with n rows and d columns, desired reduced dimensionality k  Output: Reduced-dimensional data matrix X_red with n rows and k columns  4. Sort the eigenvectors in descending order based on their corresponding eigenvalues:  sort_indices = argsort(λ)[::-1]  V_sorted = V [:, sort_indices]  5. Select the first k eigenvectors:  V_k = V_sorted [:, :k]  6. Project the original data onto V_k to obtain the reduced-dimensional data:  X_red = X_centered.dot (V_k) |

| **mRMR-based Feature Selection Algorithm 1: Implementation Steps** |
| --- |
| **Input:** $n$-dimensional original data, ${X\mathcal{\in R}}^{n}$ with $N$ samples, class labels y  **Output:** Reduced-dimensional data matrix X_red with n rows and k columns  **1** Initialize an empty set of selected features F.  **2** Calculate the mutual information between each feature and the class labels:  mi = mutual_information (X [:, i], y) # Calculate MI between feature i and y  **3** Add the feature with the highest mutual information to F:  F.add (argmax (mi))  **4** While \|F\| < k:  **a.** For each feature i not in F, calculate the relevance of i to the class labels:  rel = mutual_information (X[:, i], y)  **b.** For each feature j in F, calculate the redundancy of i with respect to j:  red = mutual_information (X[:, i], X[:, j])  **c.** Calculate the mRMR criterion for each feature i:  mrmr = rel - (1 / len(F)) * sum ([red for j in F])  **d.** Add the feature with the highest mRMR criterion to F:  F.add (argmax (mrmr))  **5** Select the features in F from the original data matrix X:  X_red = X [:, list(F)] |

1. **algorithms for MLMs**

| **The Implementation Steps for the mRMR-Based Feature Selection Algorithm 2** |
| --- |
| **Input**: $\mathrm{NumFeaturesWanted}$ is the total number of features that have been chosen., and $C$ is the set of initial features.  **Output:** $selected\_Features$ (The set of features that have been chosen)  **for** feature $f_{i}$ in $C$ do  $relevance = mutual\_Info (f_{i}, Class)$  $redundancy = 0$  **for** $feature f_{j}$ in $C$ **do**  $redundancy += mutual\_Info (f_{i}, f_{j})$  **end for**  $mrmr\_Values[f_{j}] = relevance - redundancy$  **end for**  $selected\_Features = sort(mrmr\_Values).take(NumFeaturesWanted)$ |

| **Steps to Implementing PCA-Based Feature Selection Algorithm 3** |
| --- |
| **Input**: $n$-dimensional original data, ${X\mathcal{\in R}}^{n}$ with $N$ samples and a variance threshold, $T_{\mathrm{variance}}$  **Output:** ${Y\mathcal{\in R}}^{k}$ is the reduced $k-$ dimensional data  1 Calculate the mean $\bar{X}\in\mathcal{R}^{n}$ of ${X\mathcal{\in R}}^{n}$, $\bar{X}=\frac{1}{N}\sum_{i=1}^{N} X_{i}$  2 Compute the $n\times n$ covariance matrix**,** $C_{n\times n}=\sum_{i=1}^{N} (X_{i}-\bar{X}){(X_{i}-\bar{X})}^{T}$  3 Compute the Eigen decomposition of $C_{n\times n}$ as${PDP}^{-1}$**,** where $P\in\mathcal{R}^{n}$ is matrix of Eigen Vectors and $D_{n\times n}$is the diagonal matrix with eigenvalues on the diagonal  4 Sort the Eigen vectors in descending order to find the first $k$ Eigen vectors with $\mathrm{variance}\geq T_{\mathrm{variance}}$ and create a new projection matrix, $W_{n\times k}$  5 $Y=W^{T}X$, where $Y\in\mathcal{R}^{k}$, projects data $X$ onto a new $k$-dimensional space. |

In this study, we implement MLMs to multi-class problems using the OVO approach.

| **Algorithm 4** The k-Nearest Neighbor Algorithm: Implementation Steps (k-NN) |
| --- |
| **Input**: $n$ training samples $T=\left\{ \left( X_{1},Y_{1} \right),\ldots,\left( X_{n},Y_{n} \right) \right\}$ where taking values in $\mathcal{R}^{d}\times C$,$X_{i}$ such as $i^{th}$ feature vector  $X_{i}=\left[ x_{i1}, x_{i2},\ldots,x_{id} \right]$ and $C$ a set containing class labels (0 for Diabetic (Y), 1 for Non-Diabetic (N), and  2 for Predicted Diabetic (P)), and value of nearest neighbors $k$  **Output:** The class label of unseen test data point $x$ (0 for Diabetic (Y), 1 for Non-Diabetic (N), and 2 for  Predicted Diabetic (P))  1 for each sample $X_{i}$ (current sample) from $T$, and $x_{i}$(query sample) compute the distances $d=\left( X_{i},x_{i} \right)$  2 Estimating $P\left( C \vert x \right)$ (posterior probability for each of the classes) |

| **Steps to Implementing a Decision Tree Algorithm 5 (DT)** |
| --- |
| **Input:** $n$ training samples $T=\left\{ \left( X_{1},Y_{1} \right),\ldots,\left( X_{n},Y_{n} \right) \right\}$ where taking values in $\mathcal{R}^{d}\times C$,$X_{i}$ such as $i^{th}$ feature vector  $X_{i}=\left[ x_{i1}, x_{i2},\ldots,x_{id} \right]$ and $C$ a set containing class labels (0 for Diabetic (Y), 1 for Non-Diabetic (N), and  2 for Predicted Diabetic (P))  **Output:** The class label of unseen test data point $x$ (0 for Diabetic (Y), 1 for Non-Diabetic (N), and 2 for  Predicted Diabetic (P))  1 Divide the sample $S=(i,t_{m})$ into $\acute{X}_{Left}^{(S)}$ and $\acute{X}_{Right}^{(S)}$ subsets for each sample $X_{i}$ (Based threshold $t_{m}$)  2 Compute the information gain  $G\left( \acute{X},S \right)=\frac{\left\vert\acute{X}_{Left}^{\left( X \right)} \right\vert}{\left\vert m \right\vert}H(\acute{X}_{Left}^{\left( X \right)})+\frac{\left\vert\acute{X}_{Right}^{\left( X \right)} \right\vert}{\left\vert m \right\vert}H(\acute{X}_{Left}^{\left( X \right)})$, where $H=-\sum_{C=1}^{3} P_{mC}\times\log P_{mC}$ and $P_{mC}=\frac{1}{\left\vert m \right\vert}\sum_{x_{j}\in R_{m}} I(c_{i}=C)$  3 Select the parameters $S^{*}=\underset{S}{\mathrm{argmin}} G\left( \acute{X},S \right)$ to reduce impurity to a minimum.  4. Repeat the previous steps for the subsets $\acute{X}_{Left}^{(S^{*})}$ and $\acute{X}_{Right}^{(S^{*})}$  until the depth reaches $\left\vert m \right\vert< {min}_{samples}$ or $\left\vert m \right\vert=1$ |

| **The Steps to Implementing Naive Bayes Algorithm 6 (NB)** |
| --- |
| **Input:** $n$ training samples $T=\left\{ \left( X_{1},Y_{1} \right),\ldots,\left( X_{n},Y_{n} \right) \right\}$ where taking values in $\mathcal{R}^{d}\times C$,$X_{i}$ such as $i^{th}$ feature vector  $X_{i}=\left[ x_{i1}, x_{i2},\ldots,x_{id} \right]$ and $C$ a set containing class labels (0 for Diabetic (Y), 1 for Non-Diabetic (N), and  2 for Predicted Diabetic (P))  **Output:** The most probable class label of unseen test sample $X$ (0 for Diabetic (Y), 1 for Non-Diabetic (N), and 2 for Predicted Diabetic (P))  1 Compute the $P\left( C=c_{0} \right)=\frac{\left\vert c_{0} \right\vert}{\left\vert C \right\vert}$ , $P\left( C=c_{1} \right)=\frac{\left\vert c_{1} \right\vert}{\left\vert C \right\vert}$, and $P\left( C=c_{2} \right)=\frac{\left\vert c_{2} \right\vert}{\left\vert C \right\vert}$ , which are the prior probabilities of each class.  2 Compute the posterior probability (class conditional probability) of class for the given test sample  $X=\left[ x_{1},x_{2},\ldots,x_{d} \right]$,$P\left( c_{i} \vert X \right)=P\left( X \vert c_{i} \right)P\left( c_{i} \right)=P\left( x_{1},x_{2},\ldots,x_{d} \vert c_{i} \right)= P\left( x_{1} \vert c_{i} \right)\times P\left( x_{2} \vert c_{i} \right)\times\ldots\times P\left( x_{d} \vert c_{i} \right)$  3 the final classifier for selecting the most probable class by the NB is $c_{NB}=\underset{c\in\left\{ c_{0},c_{1},c_{2} \right\}}{argmax} P\left( c \right)\prod_{i=1}^{d} P\left( x_{i} \vert c \right)$ |

| **Algorithm 7: Random Forest Implementation Steps (RF)** |
| --- |
| **Input:** $n$ training data samples $T=\left\{ \left( X_{1},Y_{1} \right),\ldots,\left( X_{n},Y_{n} \right) \right\}$ where taking values in $\mathcal{R}^{d}\times C$,$X_{i}$ such as $i^{th}$ feature vector $X_{i}=\left[ x_{i1}, x_{i2},\ldots,x_{id} \right]$ and $C$ a set containing class labels (0 for Diabetic (Y), 1 for Non-Diabetic (N), and  2 for Predicted Diabetic (P))  **Output:** The most probable class label of unseen test sample $X$ (0 for Diabetic (Y), 1 for Non-Diabetic (N), and 2 for Predicted Diabetic (P))  1 After $t$ times sampling with replacement from the previous data set, a new data set of $t$ samples (there may be duplicate samples) can be obtained. In addition, $f$ features from the n features are employed as input features using the sampling without replacement rule.  2 For the new test sample-set $X$ (with m maples and $f$ features), the Gini impurity of the subset of samples belonging to class $c_{k}$ is $C_{k}$, and the Gini impurity of the subset of samples belonging to class $c_{k}$ is:  $Gain\left( X \right)=1-\sum_{k=1}^{K} \left( \frac{\left\vert C_{k} \right\vert}{\left\vert X \right\vert} \right)^{2}$  For each feature $A$ and its possible value $a$, Compute$Gini\left( D, A \right)$ by using  $Gini\left( D, A \right)=\frac{\left\vert D_{1} \right\vert}{\left\vert D \right\vert}Gain\left( D_{1} \right)+\frac{\left\vert D_{2} \right\vert}{\left\vert D \right\vert}Gain\left( D_{2} \right)$  $D_{1}= \left\{ (\vec{x},y)\in D\vert\vec{x}^{A}=a \right\}$  $D_{2}= \left\{ (\vec{x},y)\in D\vert\vec{x}^{A}\neq a \right\}=D-D_{1}$  3 The optimal feature and optimal segmentation point, respectively, are A and a, which minimize Gini impurity. According to them, the training set is  separate into two sub-nodes.  4 Recursively call steps 2 and 3 for these two sub-nodes. Finally, the new data set is employed in the construction of a decision tree (with m samples and $f$  features).  5 To create an RF model, repeat the preceding steps$t$ times to create $t$ decision trees. |

| **Algorithm 8: Multiclass AdaBoost Implementation Steps (AdaBoost.MH)** |
| --- |
| **Input:** $\left\{ \left( x_{1},Y_{1} \right),\ldots,\left( x_{n},Y_{n} \right) \right\}$, where $x_{i}\mathcal{\in X,}y_{i}\mathcal{\in Y}$  **Output:** The final hypothesis  1 Initialize: $D_{1}\left( i\mathcal{,l} \right)=\frac{1}{nk}$  2 **For** $t=1,2,\ldots,T:$  Train weak learner using distribution $D_{t}$  Get weak hypothesis $h_{t}\mathcal{:X\times Y}\mathbb{\to R}$  Chose $\alpha_{t}\mathbb{\in R}$  Update:  $D_{t+1}\left( i\mathcal{,l} \right)=\frac{D_{t}\left( i\mathcal{,l} \right)e^{\left( -\alpha_{t}Y_{i}\mathcal{[l]}h_{t}\left( x_{i}\mathcal{,l} \right) \right)}}{Z_{t}}$ , where $Z_{t}$ is normalization factor (chosen so that $D_{t+1}$ will be a distribution)  3 Output the final hypothesis:  $H\left( x\mathcal{,l} \right)=Sign\left( \sum_{t=1}^{T} \alpha_{t}h_{t}\left( x\mathcal{,l} \right) \right)$ |

| **Algorithm 9: Multiclass Support Vector Machine Implementation Steps (SVM)** |
| --- |
| **Input:** $n$ training data samples $T=\left\{ \left( X_{1},Y_{1} \right),\ldots,\left( X_{n},Y_{n} \right) \right\}$ where taking values in $\mathcal{R}^{d}\times C$,$X_{i}$ such as $i^{th}$ feature vector $X_{i}=\left[ x_{i1}, x_{i2},\ldots,x_{id} \right]$ and $C$ a set  containing class labels (0 for Diabetic (Y), 1 for Non-Diabetic (N), and 2 for Predicted Diabetic (P))  **Output:** The most probable class label of unseen test sample $X$ (0 for Diabetic (Y), 1 for Non-Diabetic (N), and 2 for Predicted Diabetic (P))  1 Define:   - 1. $CandidateSV=\left\{ closest pair from opposite classes \right\}.$   2. $S stands for Support Vector Set.$   3. $\alpha_{i} are the corresponding coefficients in kernels function associated with the Support Vectors.$   4. $p\in S (a point).$   5. $c is \mathrm{blocks}.$   2 **while** there are violating points **do**  Find a violator  $CandidateSV=CandidateSV\cup Violator$  **if** any $\alpha_{p}<0$ due to addition of $c$ to $S$ **then**  $CandidateSV=CandidateSV\backslash p$  repeat till all such points are pruned  **end if**  **end while** |
